# Micro and nanoscale probes for spatiotemporal cell analysis and manipulation

**DOI:** 10.1039/d6an00915h

**Published:** 2026-10-06

**Authors:** Henry F. S. Budds, Wellydo Escarião, Richard W. Taylor, Vahid Sandoghdar, Chalmers C. C. Chau, Sheena E. Radford, Ciro Chiappini, Eric W. Hewitt, Paolo Actis

**Affiliations:** a School of Electronic and Electrical Engineering, University of Leeds Leeds UK p.actis@leeds.ac.uk; b Bragg Centre for Materials Research, University of Leeds Leeds UK; c Astbury Centre for Structural Molecular Biology, University of Leeds Leeds UK; d Centre for Craniofacial and Regenerative Biology, King's College London London UK; e Max Planck Institute for the Science of Light Erlangen Germany; f Max-Planck-Zentrum für Physik und Medizin Erlangen Germany; g School of Molecular and Cellular Biology, University of Leeds Leeds UK; h London Centre for Nanotechnology, King's College London London UK

## Abstract

Understanding dynamic cellular processes requires technologies capable of probing living cells with high spatial and temporal precision while preserving viability. Conventional approaches, including bulk omics, microinjection, and electroporation, often provide only static snapshots or induce significant cellular perturbation. Recent advances in micro- and nanoscale probes are enabling minimally invasive intracellular delivery, sampling, sensing, and manipulation with unprecedented precision. This review examines the current state of the art in micro and nanoscale probe technologies, including fluidic force microscopy (FluidFM), nanopipettes, open-space microfluidic probes, and nanoneedle arrays. We discuss how these complementary platforms enable controlled delivery of biomolecules, longitudinal molecular sampling, intracellular electrophysiology, electrochemical sensing, and spatiotemporally resolved analyses from single cells to living tissues. We placed particular emphasis on their ability to overcome the limitations of destructive endpoint assays by facilitating repeated interrogation of the same cell, quantitative molecular delivery, and multimodal measurements with subcellular resolution. Finally, we discuss the remaining engineering challenges, including improving throughput, integrating complementary sensing modalities, and advancing these promising technologies into robust platforms for laboratory studies of cultured cells and tissues.

## Abstract 2

This review explores how micro- and nanoscale probes enable minimally invasive delivery, sampling, sensing, and manipulation in living cells with high spatial and temporal precision.

## Graphical abstract

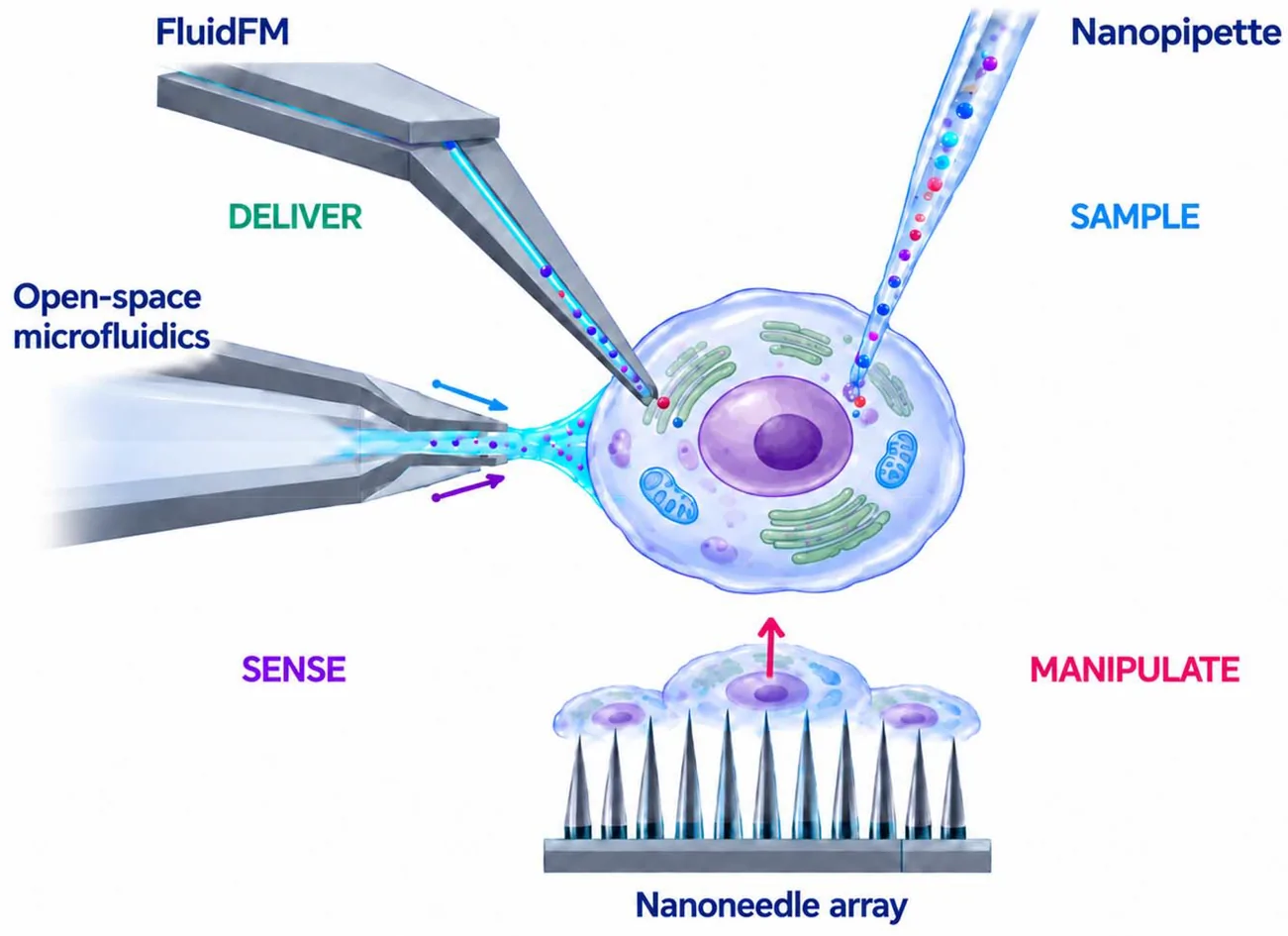

## 1. Introduction

Biological processes such as development, immunity, cellular differentiation, and disease progression unfold through continuous interactions across space and time, yet current technologies largely provide static snapshots that obscure these dynamic mechanisms. Rather than relying on destructive techniques, emerging approaches allow scientists to access, sample, and perturb intracellular environments with single molecule precision while preserving cell viability. Although still in their infancy, these technologies are empowering the biological community to explore dynamic molecular analyses, from single cells to living tissues with unprecedented spatiotemporal resolution.

Conventional methods of introducing cargo into cells, such as electroporation and microinjection, are often limited by poor uptake efficiencies and significant cell perturbations. Microinjection can cause changes in the F-actin cytoskeleton, cell rounding, and cell death.^1,2^ Nanoprobes can be used to introduce various cargo (including nanoparticles, dyes, RNA, DNA, and proteins) into the nucleus or cytoplasm at defined timepoints and to study the cellular effects of the delivered cargo. In addition, living cells can be sampled repeatedly over time, providing a dynamic view of the genotype of interest rather than a static snapshot at a single time point.^3–5^ Progress in this area is also driving the refinement of sample preparation protocols for downstream analyses and biotech companies are starting to commercialise amplification kits compatible with sub-single cell amounts of RNA to generate high-quality RNA-seq datasets. The progress is by no means limited to the analysis of nucleic acids, and nanotechnology approaches are being combined with structural biology techniques, such as cryo-electron microscopy (cryo-EM) and advanced mass spectrometry techniques.^6–8^

Throughout this review, we use the terms “micro and nanoscale probes” to encompass various technologies that establish localised physical or chemical interfaces with living cells, including nanopipettes, nanoneedles, FluidFM cantilevers, and open-space microfluidic probes. While these platforms differ substantially in geometry and operating principles, they share the common objective of enabling minimally invasive spatiotemporal access to cellular processes. We aim to provide the reader with a technological framework spanning approaches from the use of single probes to access a cell of interest to approaches where cultured cells and tissues are interfaced on arrays of nanoscale probes and discuss the associated trade-offs in throughput and resolution across methodologies.

## 2. Fluidic force microscopy

### 2.1 Intracellular delivery by FluidFM

Fluidic force microscopy (FluidFM) is an adaptation of atomic force microscopy (AFM), a scanning probe microscopy technique which employs a nm-size tip to map the topography of materials with nanoscale resolution. In 2009, Meister *et al.* first described FluidFM, where a microfluidic channel was incorporated into a cantilevered AFM tip.^9,10^ By applying a pressure within the microchannel, a cargo can be ejected from the AFM tip and the amount delivered can be controlled by the duration and magnitude of the applied pressure.

FluidFM probes can be precisely positioned over a cell in culture using automated force feedback. The amount of force applied to the cell can be precisely controlled, allowing the FluidFM tip to mechanically penetrate the cell membrane. This approach allows injection with subcellular resolution by controlling positioning and force applied, enabling controlled cytoplasmic or nuclear injection.^9,11^ Alternatively, a FluidFM tip can be used to apply gentle contact to the cell surface, where specific cargo such as membrane-permeable dyes can be delivered to the cell surface.^9^

FluidFM probes can be fabricated with a range of tip geometries and pore diameters, allowing the probe design to be tailored to the specific application and cargo size. For example, the first FluidFM probe described had a ∼250 nm pore,^9^ whereas probes for cell injection that are currently available commercially have a ∼600 nm pore,^12^ although custom probes have been reported with a pore as small as 5 nm.^13^

FluidFM has been used in the injection of mRNAs, plasmids, and small extracellular vesicles (sEVs) into HeLa cells.^14^ Immediately following injection, sEVs were initially observed in a small area of the cytoplasm, presumably close to the site of injection, but within one hour, had dispersed more widely in the cytoplasm. In contrast to spontaneously internalised sEVs, which were primarily taken up *via* endocytosis and then degraded, FluidFM-injected sEVs evaded lysosomal degradation. Longitudinal observations revealed that sEVs localised near the cytoplasmic face of plasma membranes of both the injected cell and neighbouring cells, suggesting that they were also transferred between cells.

In addition, FluidFM has been used to deliver CRISPR components into the nucleus of Chinese Hamster Ovary (CHO) cells to generate single and multiple knockout monoclonal cell lines.^15^ CHO cells are commonly used in industry as a standard mammalian cell line for the large-scale production of recombinant proteins including monoclonal antibodies and therapeutics. Direct nuclear delivery of CRISPR components reduced the timeframe of generating a monoclonal CHO cell line from the industry standard of >10 weeks to 2–3 weeks. This demonstrates an application for FluidFM in the generation of cellular models, as well as potentially streamlining the production of protein-based therapeutics.

A particularly exciting recent application of FluidFM is the investigation of endosymbiosis and host–pathogen cell interactions through the direct injection of intact bacteria into the cytoplasm of mammalian^17,18^ and fungal cells.^19–21^ This method highlights new opportunities for synthetic strategies aimed at designing endosymbiotic systems with desired traits. Implantation of mammalian cells with bacteria was performed with 1.2–1.6 µm microchannel diameter FluidFM tips with altered pore geometry by focused ion beam milling. Fluorescence microscopy allowed for the observation and tracking of fluorescently labelled bacteria within the FluidFM microchannel before injection and inside the host cell's cytoplasm after injection, showing that bacteria were able to move and replicate within certain host cells.

In 2026, Zare-Eelanjegh *et al.* used FluidFM probes to apply a mechanical stimulus *via* indenting or puncturing the surface of HeLa cells alongside injection of an actin polymerisation inhibitor.^16^ The stimulation was followed by Fluorescence Lifetime Imaging Microscopy (FLIM), where the change in fluorescence lifetime of an endoplasmic reticulum (ER) specific membrane tension probe was used to report changes in membrane tension. Integrating FluidFM with FLIM enabled real-time, high-resolution mapping of intracellular tension dynamics during precise delivery while maintaining a constant mechanical stimulus (Fig. 1A–H).

**Fig. 1:**
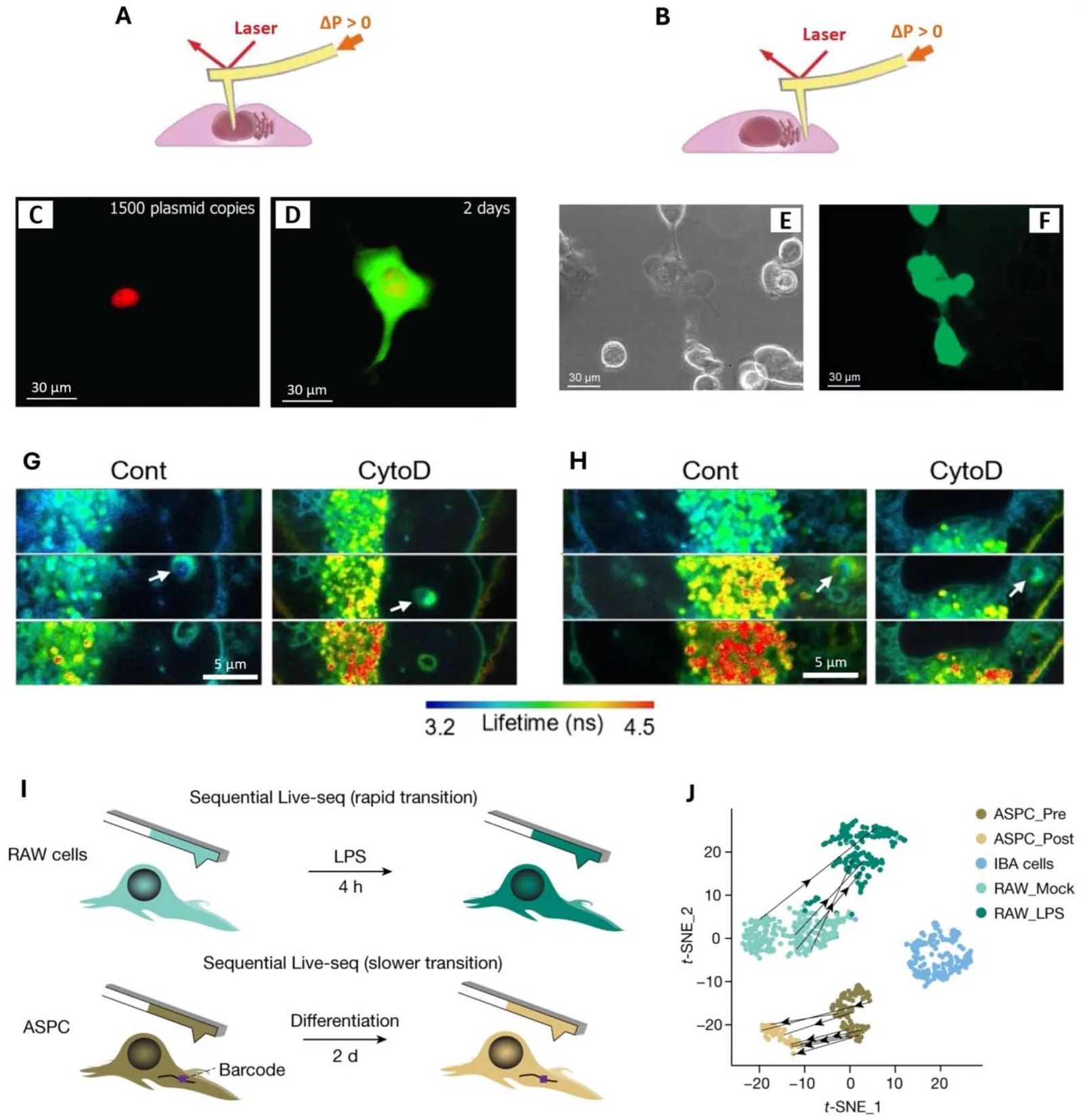
FluidFM enables nanoinjection and nanobiopsy of single cells. (A and B) FluidFM allows for the injection of cargo into the (A) nucleus and/or (B) cytoplasm *via* mechanical penetration of the cell membrane with a cantilevered FluidFM probe. The force applied to the cell can be calculated by measuring the deflection of the cantilever using a laser, represented by the red arrow, and a photodetector. Flow of liquid into the cell is induced *via* an applied pressure, *P*, within the microchannel. (C and D) Nuclear injection using FluidFM was first demonstrated with the coinjection of HeLa cell nuclei with (C) DexTRITC and (D) the plasmid pmaxGFP, resulting in successful transfection and expression of GFP. (E and F) Cytoplasmic injection by FluidFM was first described with the injection of FITC into the cytoplasm of NG108 neuroblastoma cells. (G and H) A recent application of FluidFM simultaneously injected the (G) nucleus or (H) cell periphery of HeLa cells with DMSO or cytochalasin D (CytoD) while mechanically perturbing the respective subcellular region with defined force. This was paired with fluorescent lifetime imaging microscopy (FLIM) to map intracellular tension dynamics before (top), during (middle), and after (bottom) mechanochemical stimulation. (I and J) FluidFM probes for sequential transcriptomic analysis. (I) RNA can be extracted from single cells sequentially, including before and after lipopolysaccharide (LPS) stimulation of monocyte or macrophage-like RAW264.7 cell line (RAW) or adipogenic differentiation of primary mouse adipose stem and progenitor cells (ASPCs). (J) Visualisation of cell transcriptomic transition *via t*-distributed stochastic neighbour embedding (*t*-SNE) projection map of integrated Live-seq and scRNA-seq data. (A), (B), (G), and (H) are reproduced from ref. 16, used under Creative Commons CC BY 4.0 license. (C) and (D) are reproduced from ref. 11 with permission from WILEY-VCH Verlag GmbH & Co. KGaA, Weinheim, © 2012. (E) and (F) are reproduced from ref. 9 with permission from American Chemical Society, © 2009. (I) and (J) are reproduced from ref. 5, used under Creative Commons CC BY 4.0 license.

### 2.2 Live-seq

Chen *et al.* established Live-seq in 2022, a pioneering technique based on FluidFM, allowing for pressure-driven RNA extraction from living cells at distinct time points and the repeated sampling of RNA from a single cell over time.^5,22^ As a proof of concept, the authors mapped a cell's trajectory by sequentially profiling the transcriptomes of individual macrophages before and after lipopolysaccharide (LPS) stimulation, and of adipose stromal cells pre- and post-differentiation. The authors also demonstrate that Live-seq can function as a transcriptomic recorder by preregistering the transcriptomes of individual macrophages that were subsequently monitored by time-lapse imaging after LPS exposure (Fig. 1I–J).

This work built upon a seminal paper from 2016 in which Guillaume-Gentil *et al*. showed the potential of the FluidFM for performing single cell biopsies.^22^ The authors demonstrated a biopsy of 0.1–3.2 pL from living cells, followed by molecular and structural analyses of the extracted material with negative stain TEM, enzymatic assays, and qPCR-based gene expression profiling.

A recent creative application of FluidFM involved the development of mitochondrial “transplantation”, where the extraction, injection, and transplantation of organelles into and from living cells was achieved with subcellular precision.^23^ Transplanting both healthy and drug-impaired mitochondria into primary keratinocytes made it possible to monitor the recovery of mitochondrial subpopulations. The transplanted mitochondria began fusing with the recipient cell's mitochondrial network within 20 minutes, and this process continued for more than 16 hours. Following transfer and subsequent cell divisions, donor mitochondrial DNA (mtDNA) replicated in recipient cells without requiring selection pressure. This approach offers new opportunities to study organelle physiology and homeostasis.

## 3. Nanopipette-based approaches

### 3.1 Single-cell nanoinjection

An alternative approach to AFM for controlled cell manipulation relies on the scanning probe technique scanning ion conductance microscopy (SICM). SICM employs nanopipettes for non-contact topographical mapping of surfaces in solution^24,25^ and it has also been employed for voltage-driven single cell injection.^26–28^ Nanopipettes are easily fabricated from quartz capillaries into needle-like probes with a nanopore at the tip, which acts as an interface between two liquid environments. Applying a potential difference across the two electrolyte solutions induces an ionic current which changes depending on the proximity of the nanopipette tip and the surface, enabling the automated positioning of the nanopipette near the cell of interest.

Nanopipettes can also function as single molecule sensors. When individual molecules pass through the nanopore at the tip, they can cause temporary changes in the measured ionic current. These temporary modulations can be analysed to provide information about the size, shape, and charge of each molecule as well as the number of molecules being ejected through the pore.^29–34^

A recent development is the combination of nanopipettes both as single molecule sensors and as a nanoinjection tool to perform single molecule resolution delivery into cells. This nanoinjection approach has enabled the quantitative delivery of a range of species into the cytoplasm or nucleus. These include fluorescent probes, nanoparticles, DNA, as well as globular and fibrillar proteins^29,35^ (Fig. 2).

**Fig. 2:**
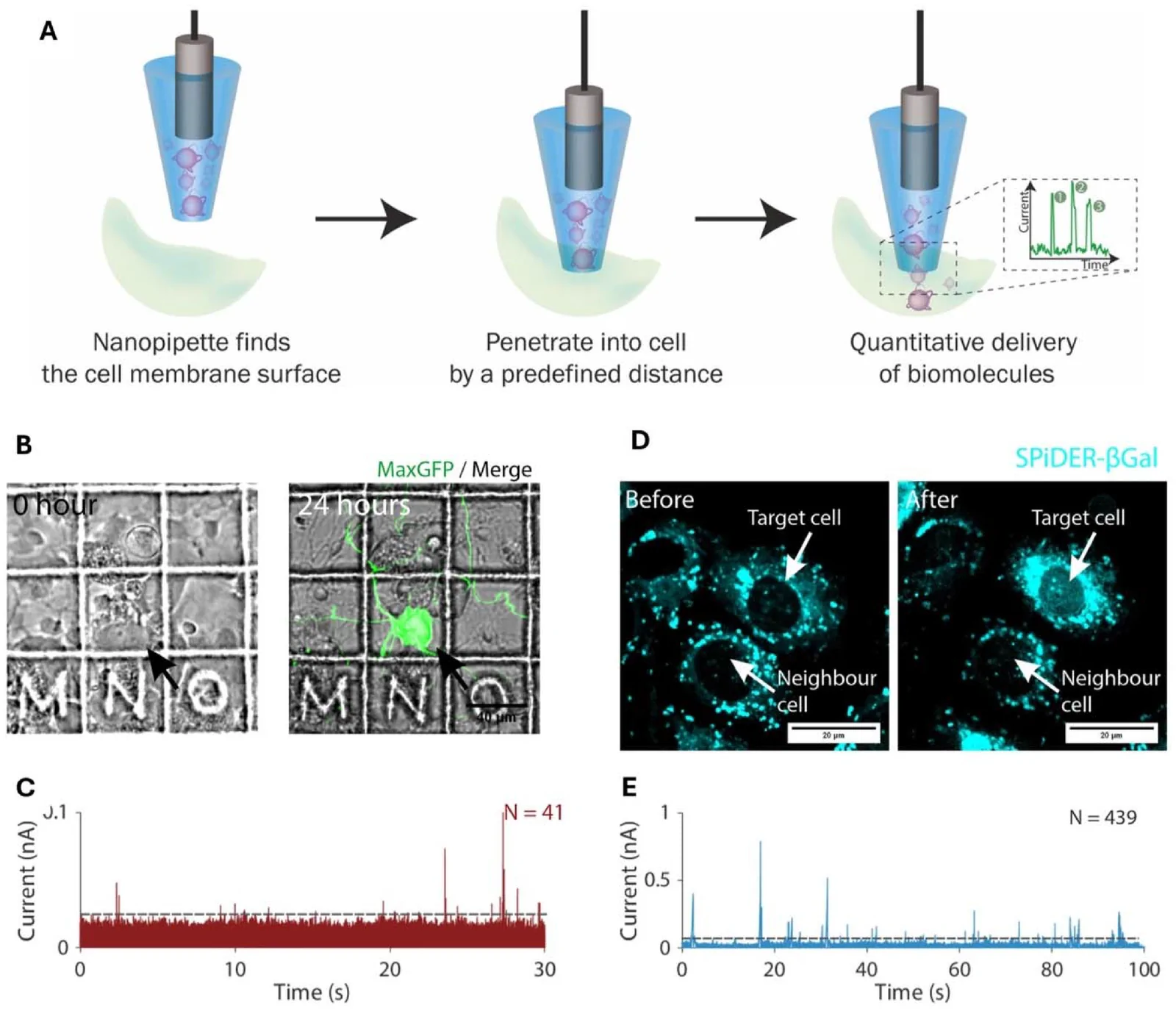
Quantitative nanoinjection of single cells by SICM-guided nanopipettes. (A) Nanopipettes can be used as a scanning probe in scanning ion conductance microscopy (SICM), where the proximity of the nanopipette tip to a surface causes a change in induced ionic current. This enables imaging of cell topography as well as precise positioning for quantitative nanoinjection. Nanopipettes can be positioned above a cell and lowered further until it penetrates the cell membrane (determined by moving a predefined distance or by measuring change in ionic current). Application of a suitable voltage then triggers delivery of charged cargo into the cytoplasm/nucleus, where measured current disruptions can be used to detect the number of molecules delivered into the cell. (B) and (C) Quantitative nanoinjection using a 24 nm nanopipette was performed on a primary dorsal root ganglion (DRG) neuron, where the (C) respective current trace confirmed a total of 41 pMaxGFP plasmids were delivered into the nucleus. (B) Successful transfection and expression of GFP was confirmed by imaging 24 h following nanoinjection. (D) and (E) The same quantitative nanoinjection approach was used to inject (E) 439 β-galactosidase proteins into the nuclei of HUVEC cells, (D) where β-galactosidase enzymatic activity was detected by imaging using a membrane-permeable fluorescent substrate SPiDER-βGal. Reproduced from ref. 29, used under Creative Commons CC-BY 4.0 license.

One intriguing observation is that the sensitivity and signal-to-noise ratio (SNR) of nanopipette sensing is enhanced during delivery into the intracellular environment when compared to an electrolyte bath.^29^ This indicates that the intracellular environment, potentially *via* macromolecular crowding, contributes to the increased SNR and enhancement of the sensitivity for the detection of macromolecules by the nanopipette when used in cells.

Single molecule nanoinjection will enable precise dose response studies because the number of delivered molecules can be measured with single molecule precision. However, each cell must be injected individually, meaning the throughput is low at ∼10 cells per hour.^29^

Nanoinjection is associated with better cell viability *in vitro* 24 h following injection when compared to microinjection (85–92% using a 100 nm nanopipette *versus* 36–42% viability using a 500 nm micropipette), osteosarcoma cell line U2OS was not significantly reduced for applied voltages of up to 1.0 V for 1 min when using a 100 nm nanopipette, a significant reduction was observed when applied for 5 min indicating that careful selection of the magnitude and duration of the applied voltage is essential to maintain high cell viability. Similarly, 85% of U2OS cells nanoinjected for 5 min at 500 mV were viable 24 h post injection, but this was reduced to 49% for cells nanoinjected for 5 min at 1 V.^2^ Conversely, Chau *et al.* reported no pronounced change in cell viability after 24 h using 25 nm nanopipettes with immortalised HeLa cells and primary dorsal rat ganglion neurons,^29^ which may suggest using nanopipettes smaller than 100 nm in diameter could further improve cell viability.

### 3.2 Nanoelectroporation

In 2025, Marcuccio *et al.* developed an alternative SICM-based nanoelectroporation approach which relies on localised electroporation by applying voltage pulses through single and double-barrelled nanopipettes.^36^ The authors have shown that the number of applied voltage pulses was linearly associated with fluorescent intensity of the cell following delivery of the cargo dextran-FITC (Fig. 3). Moreover, this approach enabled the controlled delivery of both charged and neutral biomolecules into subcellular compartments. This contrasts with nanoinjection, which relies on the nanopipette penetrating the membrane of the cell and the cargo molecules being charged.

**Fig. 3:**
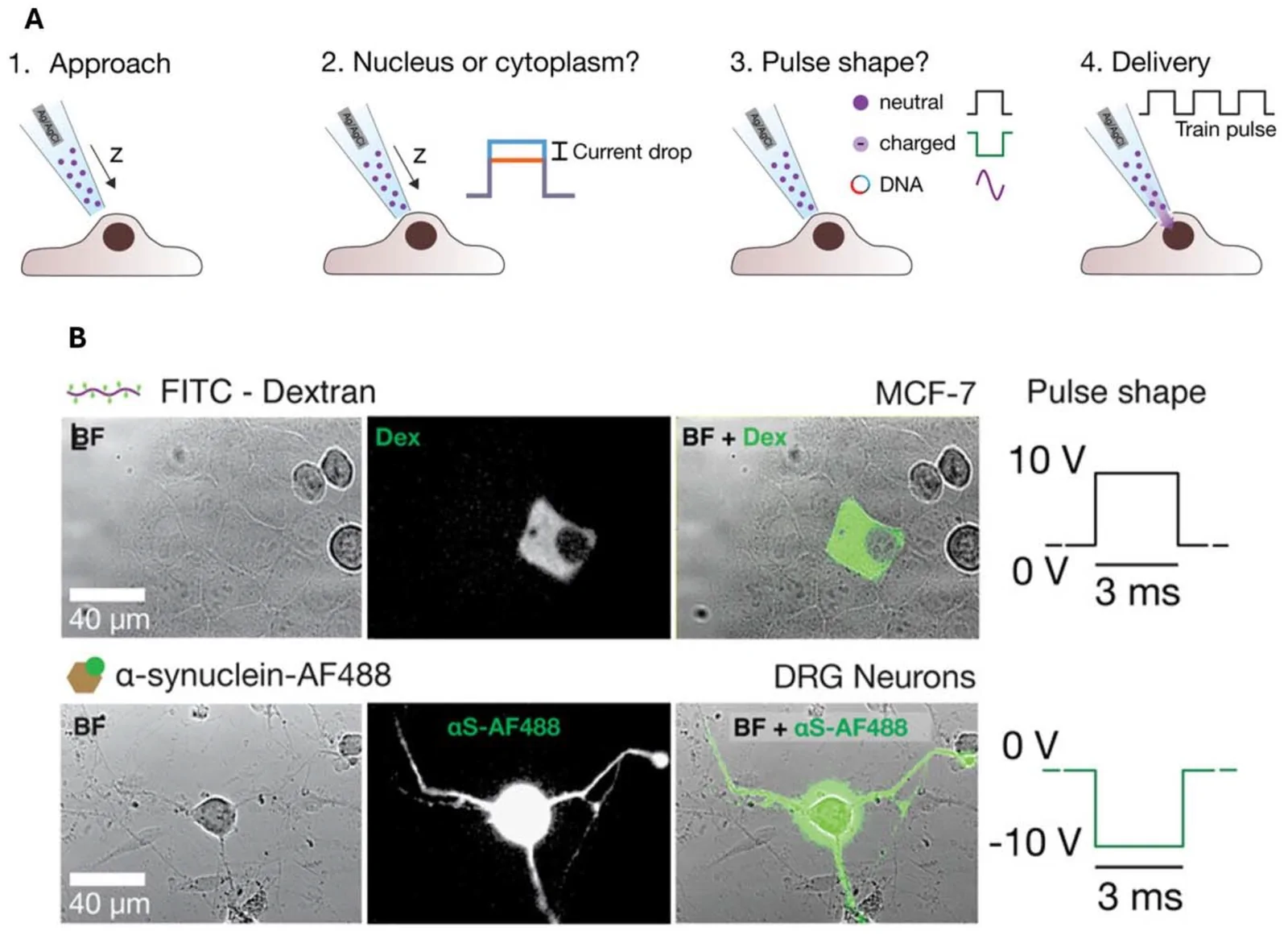
Localised nanoelectroporation allows single molecule delivery into cells without mechanical disruption of the cell membrane. (A) Nanopipettes can be used to approach the cellular/nuclear membrane until a predefined current drop is achieved, confirming positioning for localised delivery without mechanically penetrating the cell membrane. The application of different voltage pulses can be used to locally electroporate and deliver differently charged cargo into the nucleus and/or cytoplasm. (B) Using different pulse shapes, differently charged cargo can be delivered into cell *via* localised nanoelectroporation, including neutrally charged FITC-Dextran into MCF-7 cells *via* a positive voltage pulse and negatively charged AF488-tagged α-synuclein monomers into rat primary dorsal root ganglion (DRG) neurons using a negative voltage pulse. Reproduced from ref. 36, used under Creative Commons CC-BY 4.0 license.

Alternative approaches for cell nanoelectroporation include the use of the nanofountain probe electroporation system (NFP-E), an automated electroporation system recently utilised for CRISPR/Cas9 gene editing.^37^ NFP-E is a single-cell electroporation method that was combined with a deep convolutional network and a cell–nanopipette contact algorithm to determine the location of a cell and then position the probe over the cell for the application of electroporation pulses. The automated NFP-E was combined with microconfinement arrays for the culture of individual cells to enable the delivery plasmids, proteins, and Cas9-ribonucleoprotein complexes for gene editing into hard-to-transfect cells.^37–39^

### 3.3 Single-cell nanobiopsy using nanopipettes

Marcuccio *et al.* developed a nanobiopsy platform technology enabling the extraction of femtoliter volumes of cytoplasm and the simultaneous injection of exogenous molecules into individual cells *via* a double barrel nanopipette.^3^ The nanobiopsy approach relies on electrowetting rather than pressure to drive the extraction of cellular content.^40^ Marcuccio *et al.* employed a double-barrel nanopipette to sequentially extract cytoplasmic content with one barrel while nanoinjecting a fluorescent tag with the second barrel for longitudinal tracking. The capability to nanoinject exogenous molecules alongside nanobiopsy considerably broadens the scope of applications for the technique, as it could be further integrated with molecular labelling and gene editing. The authors demonstrated the multigenerational longitudinal nanobiopsy of the same cell (and its progeny) to profile gene expression changes over 72 hours. As a proof of concept, they investigated changes in gene expression in a cell culture model of glioblastoma, the most aggressive brain cancer, demonstrating sampling of the same cell and its progeny longitudinally throughout exposure to chemo- and radiotherapy (Fig. 4A and B). The nanobiopsy technology enabled the determination of transcriptomic changes that may underpin the ability of these cancer cells to adapt and resist treatment.^3^

**Fig. 4:**
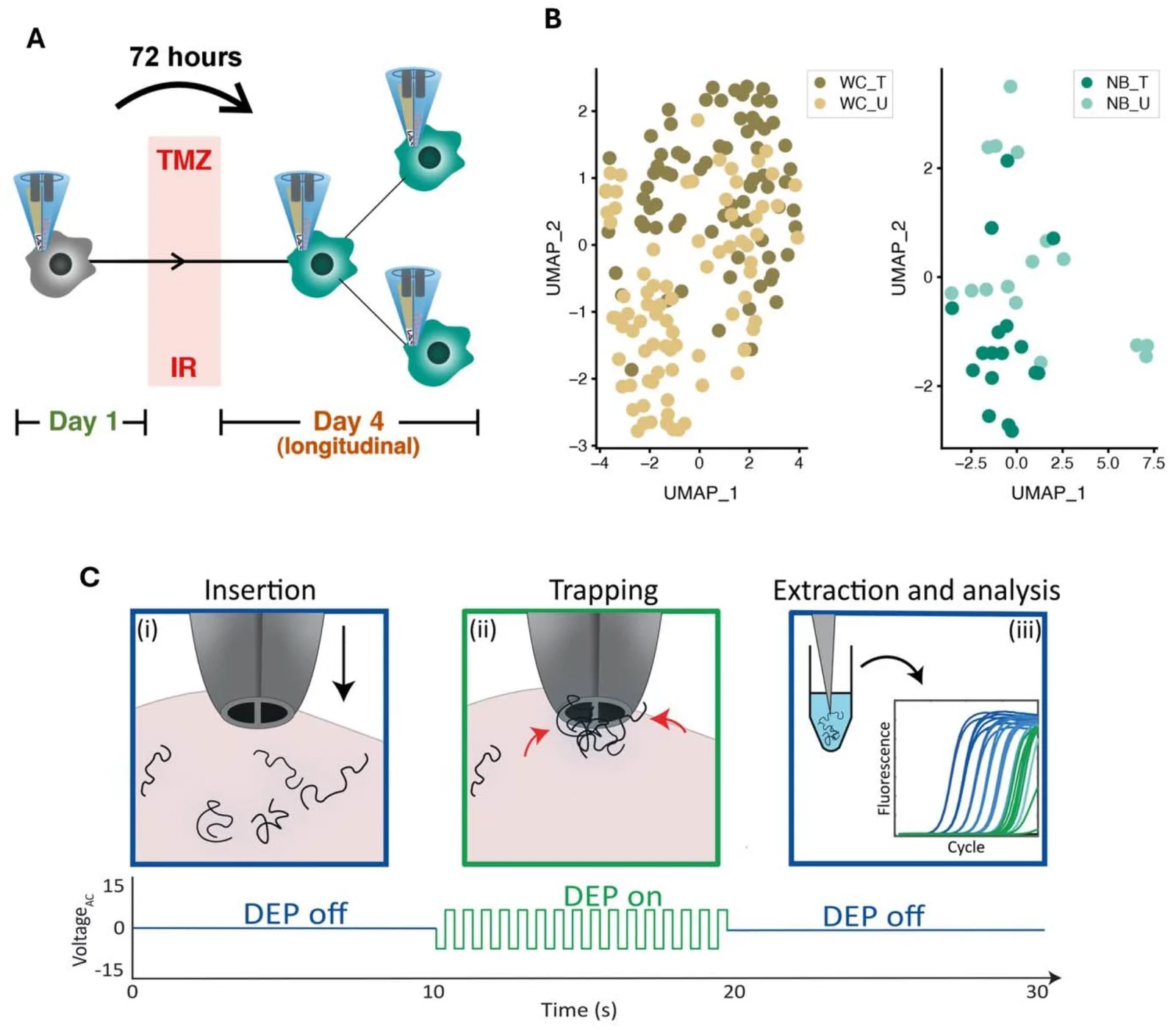
Nanopipettes and nanotweezers for single cell biopsy. (A) Sequential nanoinjection and nanobiopsy of glioblastoma cells (and their progeny) before and after treatment with chemotherapeutic (temozolomide (TMZ)) and irradiation (IR). (B) Change in transcriptomic signature following treatment visualised *via* Uniform Manifold Approximation and Projection (UMAP). Treated and untreated whole-cell lysate (WC_T and WC_U respectively) and longitudinal nanobiopsy samples (NB_T and NB_U respectively). (C) Nanotweezers, fabricated by carbon deposition on the tip of double-barrelled nanopipettes, can be used to extract RNA, DNA, protein, and single organelles from cells *via* dielectrophoretic trapping. Nanotweezers were recently used to detect the localisation of mRNA and extract RNA for gene expression analysis from primary rat hippocampal neurons in culture. (A) and (B) are reproduced from ref. 3, used under Creative Commons CC-BY 4.0 license. (C) reproduced from ref. 43, used under Creative Commons CC-BY-NC 3.0 license.

Nanobiopsy can also be used for the direct extraction and evaluation of intraluminal vesicles (ILVs). Ida *et al.* showed the direct collection of CD63(+) multi-vesicular bodies in ILVs from HEK293 CD63-pHluorin-RFP cells and showed that their RNA expression profiles were different from that of cytosol and extracellular vesicles isolated by ultracentrifugation.^41^ The authors demonstrated that hsa-miR-145-5p was mainly detected in ILVs, barely detected in cytosol, and present in 1/3 of extracellular vesicles. Thus, nanobiopsy allowed for direct assessment of the local accumulation of miRNAs in cells, demonstrating the potential of nanobiopsy for spatiotemporal sampling within living cells.

The nanobiopsy approach can also be employed to sample RNA from tissues. Stanley *et al.* extracted RNA from 22 individual cells within a breast cancer tissue slice using a robotic nanopipette platform.^42^ The mRNA from the aspirate was then selectively captured using magnetic nanoparticles and the resulting RNA-seq data provided high alignment rates (80%) with control tissue samples.

An adaption of the nanobiopsy approach was also used for the subcellular sampling of mitochondria from human tissues with a spatial resolution superior to laser capture microdissection.^44,45^ This can be employed to investigate sub-cellular mtDNA heterogeneity in conjunction with next-generation sequencing.

Slotos *et al.* have used a robotic nanopipette sampling approach to investigate the local effect of Aβ plaque-like deposits (a histopathological hallmark of Alzheimer's disease) on the spatial signature of mitochondria in a mouse microglial cell line.^46^ The study revealed that oxygen consumption is only reduced in cells close to Aβ deposits and that a distance-related gene expression pattern occurs, indicating a local initiation of mitochondrial changes in proximity of Aβ deposits.

### 3.4 Dielectrophoretic nanotweezers

Nadappuram *et al.* developed minimally invasive nanotweezers that can extract material from living cells with single-molecule resolution^47^ (Fig. 4C). These nanotweezers are based on double barrel carbon nanopipettes, which contain two closely spaced electrodes separated by gaps as small as 10–20 nm. The application of an AC voltage across the electrodes creates an electric field gradient, which allows the dielectrophoretic trapping of biomolecules.^43^ The authors demonstrated the ability to trap single molecules and extract nucleic acids from living cells for gene expression analysis without compromising cell viability. They also showed that the system can trap and extract a single mitochondrion.^47^

Sahota *et al.* also employed this technology for the extraction of individual mitochondria from live neurons and investigated the downregulation of mitochondrial genes MT-ND1 and MT-ATP6 following exposure to α-synuclein aggregates, revealing that the dysregulation was independent of the proximity of the aggregates to the sampled mitochondria.^48^ Additionally, Sahota *et al.* applied nanotweezers for mapping RNA compartmentalisation in the soma and dendrites of hippocampal neurons at different stages of neuronal development and tracked the relative abundance of mRNA at dendritic spine regions following stimulation of the NMDA receptor.^49^

## 4. Open-space microfluidics

Over the last two decades, open-space microfluidics has emerged as a powerful strategy for precise, minimally invasive manipulation of live cells.^50^ One of the earliest and most established embodiments of this concept is the open channel, in which cells are confined and accessed *via* a structured, fixed-geometry conduit left exposed to the surrounding environment rather than sealed within a closed device. This approach was first demonstrated by Hsu *et al.*, who fabricated open-air “microcanals” enabling direct micropipette access for patch-clamp recording and intracellular injection of cultured cells.^51^ Recently, Murakami *et al.* extended this concept by combining deterministic single-cell trapping with automated robotic pipetting, allowing individually trapped cells to be retrieved and transferred to standard well plates for downstream single-cell assays without the random positioning and image-based picking required in unstructured arrays.^52^ Bansho *et al.* subsequently scaled this approach by two orders of magnitude, addressing the trapping yield losses caused by particle clogging (an inherent limitation of deterministic trapping) through periodically spaced detour channels that route particles around blockages to downstream sites. Arrays exceeding 10 000 traps and reproducible single-cell trapping of Jurkat cells at ∼95% efficiency were demonstrated with robotic retrieval.^53^ Taylor *et al.* extended the architectural repertoire further, introducing hydrodynamically defined virtual channels that can be reconfigured in real time, enabling directed transport, splitting, and mixing without device refabrication.^54^

The array-based and reconfigurable-channel architectures discussed above achieve “openness” by leaving a structured channel exposed; cells are confined within walls, but those walls have no roof, keeping them reachable from above. Microfluidic probes (MFPs) push this openness further still: rather than relying on any physical channel structure at all, they use hydrodynamically defined boundaries to shape a microscale liquid domain directly within an open aqueous environment, with no walls or roof of any kind. Comprising injection and aspiration apertures within a steerable mesoscopic head, MFPs enable reagents to be locally delivered, confined, and removed with micrometre precision. This freedom from fixed geometry lets the probe, rather than the cell, be the one that moves, generating a confined zone wherever it is positioned over an unstructured sample. Critically, the open architecture keeps cells accessible for parallel measurements such as optical microscopy or electrochemical sensing throughout. This has allowed MFPs to be deployed as programmable chemical scalpels, imposing defined transient biochemical states on single cells that are kinetically or spatially inaccessible by conventional means. Recently, Zhang *et al.* used such an MFP to confine short-lived free radicals to micrometre-scale regions for selective membrane stimulation at subcellular sites^55^ (Fig. 5A). Furthermore, Song *et al.* applied the technology first to control membrane wounding with real-time repair kinetics, revealing dependence on extracellular ions and glucose and pronounced cell-to-cell heterogeneity, and then demonstrated functional cargo delivery *via* localised perforation with the detergent digitonin, introducing the 13 kDa protein cytochrome c and macromolecules up to 150 kDa (FITC-dextran).^56,57^ Gao *et al.* established that perfusion micropipettes could spatially resolve oxygen consumption at single-cell resolution by exploiting flow-enhanced analyte confinement to amplify otherwise diffusion-limited signals.^58^ Zhou *et al.* extended this sensing principle by integrating enzymatic electrochemical detection into the aspiration channel to quantify real-time lactate secretion under controlled glucose perfusion, exposing orders-of-magnitude metabolic heterogeneity among neighbouring cells.^59^

**Fig. 5:**
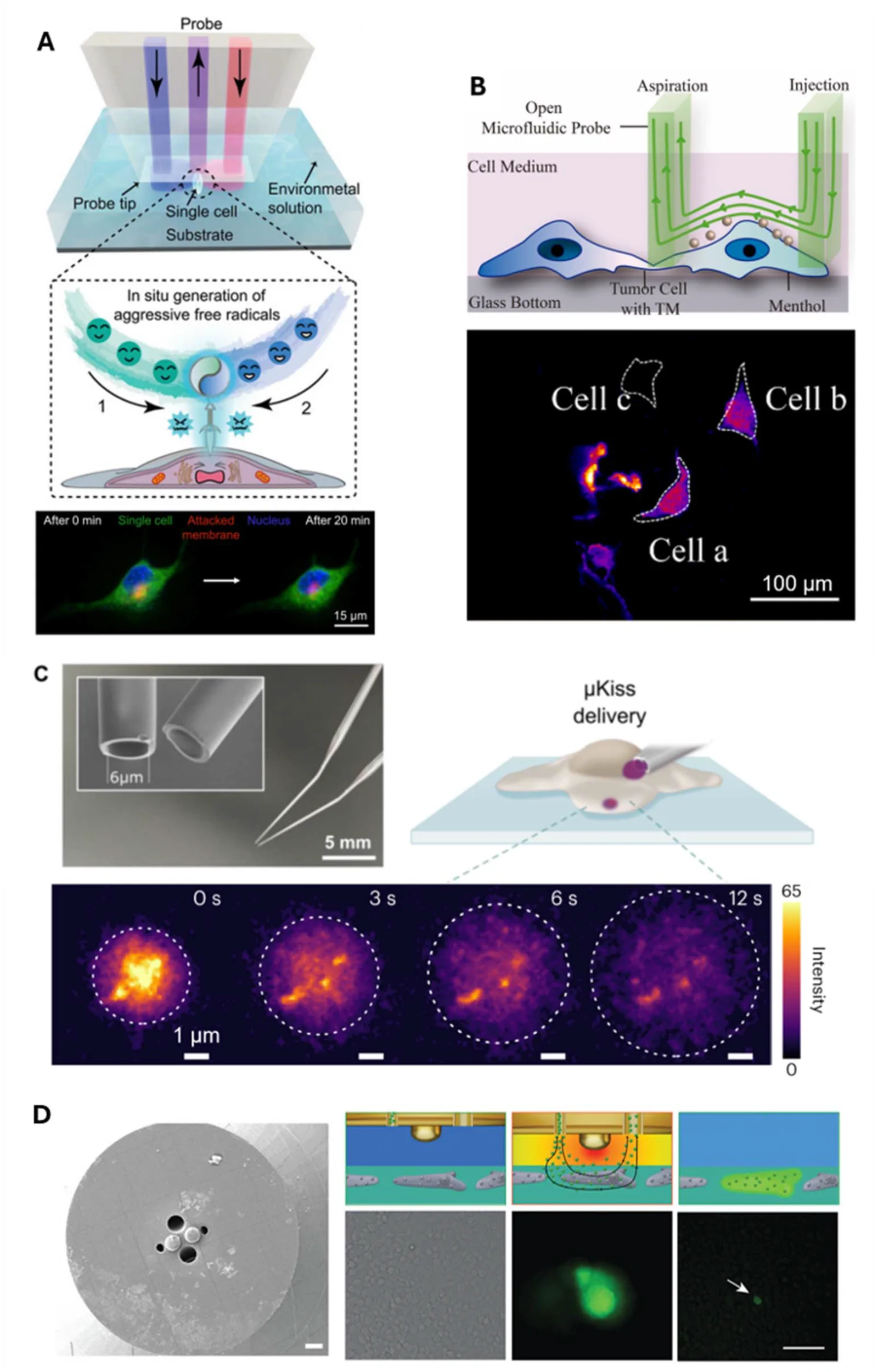
Reagent delivery to live cells in the open fluidic space. (A) Microscale chemical stimulation of a single cell *via* local *in situ* generation of free radicals. (B) Probing tumour microtubule (TM) mediated cell–cell communication through selective stimulation (200 µM menthol) of an individual cell (Cell A) and its partner (Cell B). Cell C serves a reference cell. (C) µKiss delivery of fluorescent dyes to a few-micron spot on the live cell membrane using a pair of micropipettes. Diffusion of dye molecules in the membrane is imaged over 12 s. (D) Transfection of a single cell (right; scale bar 100 µm) with a fluorescent plasmid *via* a multiphysics probe (left; scale bar 50 µm) comprising integrated microelectrodes for membrane permeabilisation and microfluidic channels for local reagent delivery. (A) reproduced from ref. 55 with permission from Wiley-VCH GmbH, © 2021. (B) reproduced from ref. 60 with permission from Elsevier B.V., © 2022. (C) reproduced from ref. 64, used under Creative Commons CC-BY 4.0 license. (D) reproduced from ref. 65, used under Creative Commons CC BY-NC-ND 4.0 license.

Beyond that of simply interrogating individual cells, Feng *et al.* used open MFPs to investigate inter-cell signal transmission by locally stimulating individual glioma cells with menthol without perturbation to neighbouring cells. The authors demonstrated directional calcium transmission and mechanically coupled responses through tumour microtubes that are normally obscured at the population level^60^ (Fig. 5B). Extending this approach to the transcriptional level, Proulx *et al.* used a multiplexed open-space platform with reagent switching under seven seconds to show that Notch pathway outputs are governed by signal duty cycle rather than total dose, establishing open-space microfluidics as a tool for dissecting how signalling information is encoded in time.^61^

Zhang *et al.* pushed the resolution of open-space microfluidics by decoupling confinement from fluid mechanics using an aqueous two-phase system. In this system unequal chemical potentials across a flowing liquid–liquid interface arrest outward diffusion, achieving sharper boundaries for subcellular stimulation than hydrodynamic confinement.^62^ A complementary strategy has seen probe geometries themselves being redesigned for more practical integration on microscope: Hu *et al.* addressed the longstanding challenge of vertical probe integration with transmission optical imaging by forming the probe head at 45°, recovering bright-field access, thus opening the possibility for implementation of additional probes and sensors into the workspace.^63^

Holler *et al.* pursued this effort of ready integration by minimising the experimental footprint as well as reducing the confined delivery volume down to the few-micron scale by developing the μKiss approach. Instead of using conventional MFP heads, this technique uses two counter-flow micropipette channels to brush reagents from a sub-femtoliter confined volume directly onto the cell membrane. The group demonstrated ∼1 μm spatial precision and 35 ms temporal resolution (Fig. 5C). The μKiss approach was used to quantify local and long-range membrane mobility and organisation as well as dynamic probing of intercellular force signalling from local perturbations.^64^ The experimental arrangement also allows integration and combination with other functionalities.

MFPs have, indeed, been integrated with complementary analytical and perturbative techniques to broaden the scope of spatially resolved live-cell interrogation. Brimmo *et al.* combined electropermeabilisation with hydrodynamic flow confinement to biopsy adherent cells in confluent culture^65^ (Fig. 5D), with Sofela *et al.* extending the approach through target gene-expression analysis and droplet compartmentalisation of retrieved cytoplasmic contents as a route toward higher-throughput single-cell analysis.^66^ At the tissue scale, Lovchik *et al.* applied MFP confinement to immunohistochemistry, reducing multistep staining protocols from hours to tens of minutes while conserving tissue and enabling multiplexed spatially selective assays.^67^

## 5. Nanoneedle arrays

### 5.1 Nanoneedle arrays for cell manipulation

Nanoneedles encompass a broad class of vertical nanostructures, including nanowires, nanopillars, nanostraws, nanotubes, and nanoneedles.^68^ They provide direct access to the intracellular space while preserving cell viability and function, enabling both interrogation and manipulation of cells, supporting biomolecular delivery, intracellular sensing, and scalable, parallelised analysis across cell populations.^68,69^

Nanoneedles are well suited for cell manipulation through the intrinsic tendency of cells to adhere, engulf, and establish a tight interface with vertical nanoprobes. They can be assembled into large arrays enabling the simultaneous analysis of hundreds to thousands of cells with minimal operator intervention.^68^ This population-scale access enables capturing biological variability, longitudinal tracking, and high-throughput biosensing applications.

A central challenge at the cell–nanoneedle interface is establishing reliable intracellular access, a prerequisite for any functional interrogation. Current evidence suggests that a dynamic interplay of transient direct access, membrane permeabilisation, and endocytic stimulation governs this interface.^68^ Insights from intracellular electrical recordings reveal that the fidelity of any intracellular readout is governed by the quality of the cell–nanoprobe interface, specifically by the combination of effective cytosolic access and tight membrane sealing.^68,70^

While nanoneedles often achieve spontaneous access during initial interfacing, this state is transient as the cells progressively exclude the probes from the cytosol.^71^ This window of access usually ranges around 10 minutes^72,73^ and can be extended to a period of 80 minutes^74^ to 24 hours^75^ when coupled with specialised optical techniques such as optoporation or optimised probe geometries. This active penetration within the cytosol relies on the forces applied to the system either externally through mechanical, electrical or optical methods or by the cells themselves.^68,70^ The tip geometry and the cellular tensegrity determine the force conditions leading to membrane breaching and intracellular access.^76–78^

Although access can be restored *via* localised nanoelectroporation at the needle tips, the effect remains transient. To improve and prolong access, nanoneedle geometries can be optimised into specialised architectures, such as nanocrowns. These nanostructures encourage the membrane to wrap tightly around the nanostructure's edge, extending the window of intracellular access by over 20-fold, as demonstrated by stable intracellular electrophysiological recordings lasting up to 1.5 hours.^73^ Alternatively, membrane permeabilisation can be achieved through the local disruption of the lipid bilayer, allowing bidirectional molecular exchange without causing permanent membrane damage.^77,79,80^ Surface functionalisation with lipophilic, amphiphilic or cationic moieties further facilitates membrane permeabilisation to achieve intracellular biomolecular exchange.^81–83^

The cell–nanoneedle interface is governed by nanostructure curvature and surface topography,^84^ which drive localised cellular activity, including mechanosensing^85^ and endocytosis.^86^ Direct visualisation using electron microscopy and 3D single-molecule super-resolution localisation microscopy has shown the recruitment of caveolae, clathrin-coated pits, and membrane wrapping around nanopillars, indicating the endocytic-like nature of passive engulfment and its dependence on nanostructure geometry^86–88^ (Fig. 6A and B). The contributions from these processes play a key role in the modality of cellular access and the fate of the delivered payload, with caveolar endocytosis identified as essential to nanoneedle-mediated injection.^82^

**Fig. 6:**
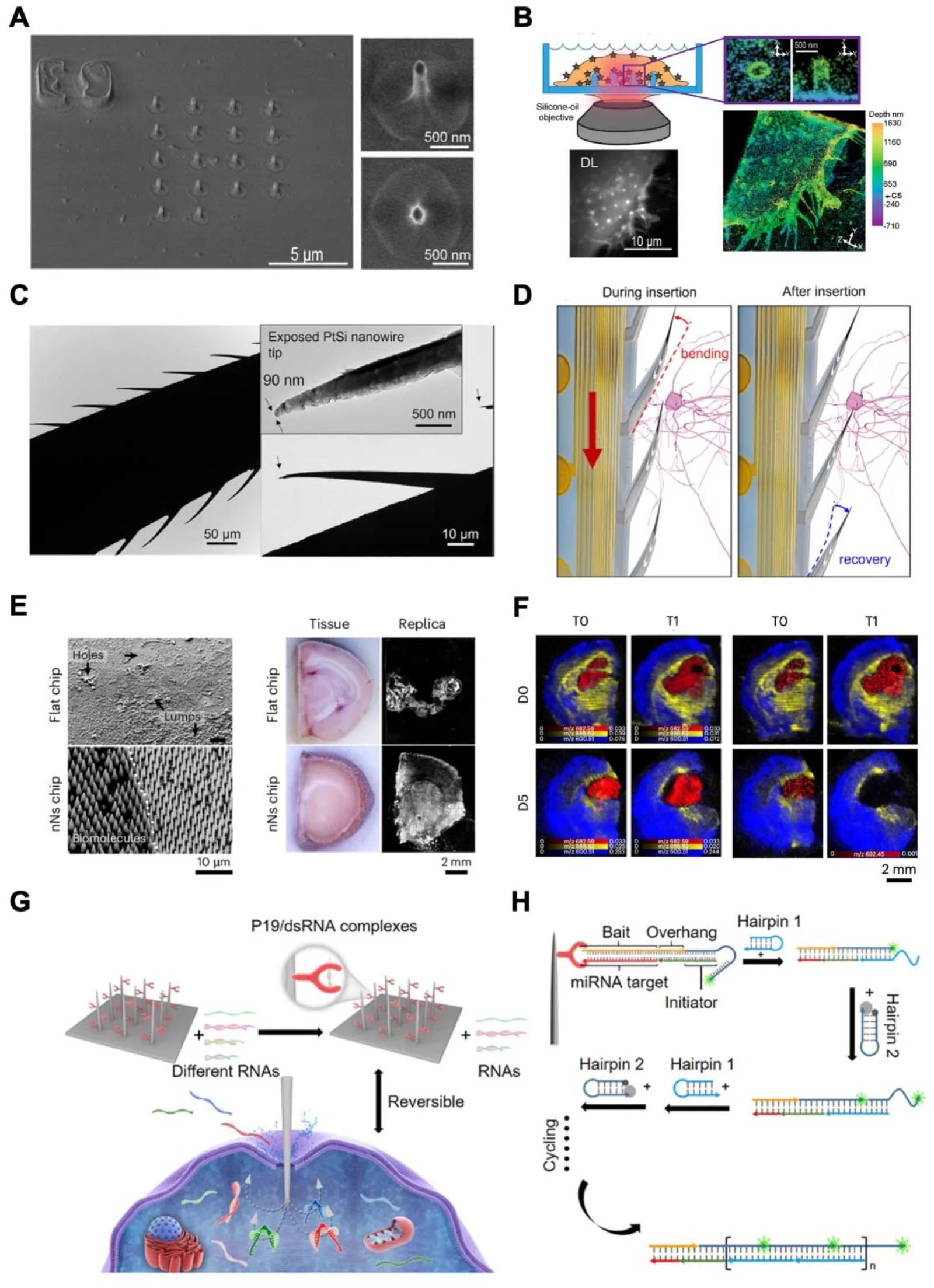
Nanoneedle arrays for cellular interrogation. (A) SEM of a patterned nanopillar array (30° tilt), with magnified views of an individual pillar showing its elliptical profile and coverslip dimple. (B) Cartoon of the imaging scheme (top left) with the corresponding diffraction-limited image below, where puncta mark nanopillar positions; on the right side, magnified views of a single pillar at different orientations, showing the membrane wrapping around it (top right), and a 3D super-resolution reconstruction revealing the membrane wrapping around the nanopillar array (bottom right). Colour encodes *Z*-position. (C) TEM images of fishbone intracellular nanowire electrode (FINE), and bright field TEM and high-resolution TEM (inset). (D) Representation of the internalisation mechanism of FINE into neurons. (E) SEM of the adsorbed molecular layer on flat *versus* nanoneedles substrates after imprinting, and photographs of the imprinted tissue and fluorescence images of the resulting molecular replica. (F) Brain slices treated (T1) and untreated (T0), at Day 0 and Day 5: images of DESI-MSI replica maps for grey matter (*m*/*z* 600.51, blue), white matter (*m*/*z* 888.62, yellow) and tumour (left panel: *m*/*z* 682.59 and right panel: *m*/*z* 692.45, red). (G) Intracellular miRNA biopsy from live cells (schematic). (H) On-needle miRNA signal amplification by hybridisation chain reaction (HCR). (A) and (B) reproduced from ref. 87, used under Creative Commons CC BY-NC-ND 4.0 license. (C) and (D) reproduced from ref. 89 with permission from Wiley-VCH GmbH, © 2025. (E) and (F) reproduced from ref. 4, used under Creative Commons CC BY 4.0 license. (G) and (H) reproduced from ref. 90, used under Creative Commons CC BY-NC 4.0 license.

Furthermore, nanoneedles can induce biophysical stimuli beyond the plasma membrane. They alter the dynamics and arrangement of actin fibres^91,92^ and intermediate filaments.^93^ They remodel the nuclear envelope and its molecular composition,^85,93^ inducing temporary breaches in the nuclear compartment and forming openings that enable uncontrolled nucleo-cytoplasmic molecular exchange without physically penetrating the nucleus.^94^ These transient openings are actively repaired by the endosomal sorting complexes required for transport (ESCRT-III) machinery, preserving cell viability while providing a window for nuclear access.^94^ Together, these data show that the biointerface is an active parameter to design nanoneedles for cellular interrogation.

Resolving intracellular physiology requires capturing both electrical activity and chemical state, which requires distinct but complementary sensing approaches. Patch clamp, the gold standard for intracellular electrical recording, achieves this by physically rupturing the membrane with a glass pipette, yielding a high-quality signal but limiting recording to single cells for short durations.^95^ Planar microelectrode arrays overcome this limitation by offering scalable, long-term extracellular recording across cell populations, but they sacrifice signal quality by recording attenuated field potentials rather than intracellular voltages.^96^ Nanostructured electrodes reconcile these trade-offs: once cell-nanoprobe contact is established, the resulting biointerface enables high-fidelity intracellular recording at a population scale without compromising cell viability.^72,97^

Nanostructures like hollow nanopillars or porous polyethylene terephthalate (PET) templates induce localised membrane wrapping to achieve the high seal resistance necessary for long-term, high-fidelity intracellular recordings that rival patch-clamp measurements and enable continuous electrophysiology longitudinal monitoring.^73,98–100^ Beyond cultured cells, nanowire arrays achieve quasi-intracellular recording in 3D intact brain tissue, showcasing the scalability of nanoelectrode-based electrophysiology in vivo.^89^

Nanoneedle electrodes also enable real-time, multi-analyte profiling of the intracellular environment, including the tracking of metabolic flux^101^ (*e.g.* ROS, NADH) and signalling molecules^102,103^ (*e.g.* H_2_O_2_, dopamine). By utilising tailored materials such as graphene oxide-wrapped gold or dual-channel nanowires, these platforms achieve label-free, cell-specific detection across both 2D cultures and complex 3D organoid models.

The platforms discussed in this section follow a common trajectory: from single-analyte measurements in isolated cell cultures to simultaneous, multimodal profiling of living tissues. The extension of nanoelectrode-based electrophysiology to 3D brain tissue^89^ (Fig. 6C and D) and the generation of spatiotemporal lipidomic maps in living tumour explants^4^ demonstrate that this transition is underway, establishing tissue-level interrogation as the next frontier of the cells-to-nanoprobes paradigm (Fig. 6E and F). Realising this potential will require addressing key challenges. Most current platforms measure only one modality at a time, and multiplexed systems capable of simultaneous electrical, electrochemical, and molecular readouts from the same cell remain limited. Integrating these modalities within a single platform without compromising biointerface integrity is an open engineering problem. Solving this will require optimisation of capture probe specificity under physiological conditions, expanding signal multiplexing capacity, and achieving the spatiotemporal resolution needed to capture dynamic biological processes in real time.

Looking ahead, overcoming the challenges associated with transient intracellular access could enable the reliable application of these techniques in cultured cells and tissues, allowing direct, real-time monitoring of disease progression and therapeutic response at the molecular level.^104^

### 5.2 Nanoneedle arrays for single cell biopsy

Nanoneedles provide direct, non-disruptive access to the intracellular space, enabling repeated extraction from living cells without compromising their viability or function^90,105^ (Fig. 6G and H). Depending on the approach, the extracted material can be collected non-specifically for broad omics profiling, yielding a bulk cytosolic sample containing all accessible molecular species,^40^ or selectively captured using surface-functionalised probes designed to isolate specific analytes.^106^ Non-specific bulk extraction can be achieved by electroporation to access the cell and drive electrophoretic migration of polyanionic species, such as RNA, through the nanoprobe into a collection reservoir.^107^ This principle was first demonstrated using alumina nanostraws as fluidic conduits into the cytosol.^78^ Conversely, targeted analysis depends on covalent or non-covalent conjugation of probes, such as aptamers or CRISPR-Cas machinery, to the nanoprobe surface, which allows specific identification of pre-defined analytes at the biointerface.^108^

The primary advantage of these nanoprobe-based conduits is the ability to perform longitudinal transcriptomic and proteomic tracking. Unlike conventional single-endpoint measurements, these techniques allow for repeated, non-destructive sampling over 72 hours or more (when coupled with electroporation or repeated transient interfacing).^107,109^ This temporal resolution is critical for resolving dynamic cellular responses and transcriptomic shifts in 2D and 3D cultures. Furthermore, another application of this strategy is demonstrated by single-cell microRNA profiling in acute myeloid leukaemia cell lines, where intracellular access is achieved through centrifugation-mediated mechanical penetration.^110^ This approach enables the multiplexed phenotyping of cellular heterogeneity that would otherwise be obscured in traditional bulk “snapshot” analyses.^110^

The expansion of these capabilities into potential clinical applications will require substantial advances in throughput and in the viability of cells and tissues subjected to these devices. For example, these technologies should be able to cross the dense extracellular matrix, stromal rigidity, and variable immune cell infiltration characteristic of solid tumours. Moreover, it will also demand a major technological shift involving the integration of nanoprobes directly into medical devices to enable molecularly selective biosensing and delivery. Progress has already been achieved as porous silicon nanoneedles have been incorporated into bioresorbable, flexible elastomers to create platforms capable of both nucleic acid delivery and extraction within a single interface.^111–114^

Building on these advancements, the field is now moving toward the generation of nondisruptive molecular imprints directly from living tissue. Mass spectrometry analysis of these imprints can resolve spatial and temporal lipidomic dynamics of living glioma tissue.^4^ This could shift the paradigm from static snapshots to longitudinal, four-dimensional profiling of living tissue. By implementing a multimodal approach using optimised nanoneedles 2D sampling with volumetric optical imaging (*e.g.*, multiphoton, light-sheet, and optical coherence microscopy), molecular identity could be co-registered onto depth-resolved tissue architecture and measured over time in their native context.^4,115,116^

Table 1 summarizes the main technical features of the platforms covered in this review focussing on spatial resolution, throughput, delivery and sampling capability, tissue compatibility and major limitations. Overall, single nanoprobe based approaches are preferred for studies focussing on precise temporal manipulation while arrayed nanoprobes offers superior performance in terms of large area spatial mapping.

**Table 1:** Comparative overview of micro and nanoprobe platforms for spatio-temporal cell analysis and manipulation. The technologies described in this review are designed for low-throughput, highly precise cellular manipulation and are therefore not intended for direct comparison with high-throughput omics workflows. Moreover, these technologies are at an early stage of development, and robust data on time to result, operator burden, and cost per sample are not yet available

Platform	Spatial resolution	Throughput	Delivery and sampling capability	Tissue compatibility & major limitations	Selected examples
FluidFM	Subcellular (1–100 nm)	Low (10 cells per day)	Pressure-driven delivery and sampling	Compatible with 2D cultured cells and thin tissues; requires specialized hollow cantilevers	Live-Seq^5^
Nanopipettes/SICM	Subcellular (1–100 nm)	Low (10 cells per day)	Voltage-driven delivery and sampling; delivery with single molecule resolution	Compatible with 2D cultured cells and thin tissues; cell viability dependent on magnitude and duration of applied voltage	Nanobiopsy^3^ and single molecule nanoinjection^29^
Open-space microfluidics (MFPs)	Subcellular to multicellular (µm–100 µm)	Moderate to high (up to 100 per experiment)	Non-contact local perfusion, stimulation and sampling	Compatible with cultured monolayers and accessible tissue surfaces; only suitable for extracellular applications	µKiss delivery^64^
Nanoneedle arrays	Subcellular to multicellular (needle pitch dependent)	Moderate to high (100–1000 cells on parallel arrays)	Parallel interfacing for delivery, sampling and sensing	Compatible with 2D/3D cultured cells and tissue slices; transient intracellular access limits longitudinal sampling	Spatiotemporal lipidomics and inCell-Biopsy^90^

## 6. Conclusions

Micro and nanoprobe technologies are rapidly advancing toward integrated platforms capable of simultaneous delivery, sensing, and sampling. These probes are redefining the landscape of single-cell analysis by enabling minimally invasive, high-resolution access to the intracellular environment. By combining precise delivery, real-time sensing, and longitudinal sampling, these technologies provide unprecedented insight into dynamic cellular processes. However, current systems often focus on a single modality, and integrating electrical, chemical, and molecular readouts within a single platform remains a significant engineering challenge. Achieving this integration without compromising cell viability or interface stability will be critical. A key challenge for longitudinal sampling is the trade-off between spatial and temporal resolution, molecular breadth, and throughput. Increasing sampling frequency or molecular coverage can compromise cell recovery, tissue viability, and spatial registration. Repeated sampling may also introduce subtle biological perturbations, particularly in fragile or patient-derived tissues, while limited molecular recovery can reduce profiling depth and increase stochastic noise. Throughput is a key limitation for nanoprobe-to-cell approaches but advances in automation, parallelisation, and machine learning-driven control are expected to address this issue. Automation and standardisation will also be essential for translating spatiotemporal analyses from proof-of-concept studies into reliable applications, requiring shared benchmarks and community-wide reporting standards.

Finally, translation beyond 2D cell cultures will require robust, scalable, and biocompatible systems capable of operating in complex biological environments. The integration of nanoprobes into medical devices and implantable systems represents a promising direction.

As the field moves toward integrated, multimodal platforms, nanoprobes are poised to play a central role in advancing both fundamental biology and precision medicine. Continued innovation in probe design, interface engineering, and system integration will be essential to fully realise their potential.

## Conflicts of interest

There are no conflicts to declare.

## Data Availability

No primary research results, software or code have been included and no new data were generated or analysed as part of this review.
